# Self-Initiated Butyl Acrylate Polymerizations in Bulk and in Solution Monitored By In-Line Techniques

**DOI:** 10.3390/polym13122021

**Published:** 2021-06-21

**Authors:** Jonas Mätzig, Marco Drache, Sabine Beuermann

**Affiliations:** Institute of Technical Chemistry, Clausthal University of Technology, Arnold-Sommerfeld-Straße 4, 38678 Clausthal-Zellerfeld, Germany; jonas.maetzig@tu-clausthal.de (J.M.); marco.drache@tu-clausthal.de (M.D.)

**Keywords:** *n*-butyl acrylate, self-initiation, free-radical polymerization, solvent influence

## Abstract

High-temperature acrylate polymerizations are technically relevant, but yet not fully understood. In particular the mechanism and the kinetics of the thermal self-initiation is a topic of current research. To obtain more detailed information the conversion dependence of the polymerization rate, *r*_br_, is determined via in-line DSC and FT-NIR spectroscopy for reactions in bulk and in solution at temperatures ranging from 80 to 160 °C. Solution polymerizations revealed that dioxane is associated with the highest *r*_br_, while aromatic solvents result in the lowest values of *r*_br_. Interestingly, *r*_br_ for polymerizations in solution with dioxane depends on the actual monomer concentration at a given time in the system, but is not depending on the initial monomer concentration. The overall rate of polymerization in bulk and in solution is well represented by an equation with three or four parameters, respectively, being estimated by multiple linear regression and the temperature as additional parameter.

## 1. Introduction

Homo- and copolymers of acrylate monomers are used in a wide variety of applications, e.g., in adhesives, coatings, and biomedical materials [1]. Despite the technical importance of these polymer materials the complexity of the polymerization mechanism is still a topic of current research. The complexity of the mechanism is caused by a large number of elementary reactions that influence the polymerization rate and the topology of the polymers. For example, inter- and intramolecular transfer reactions associated with the formation of tertiary propagating radicals and β scission of these radicals at higher temperatures are still under investigation [2,3,4,5,6,7,8,9,10,11,12,13,14].

Industrially, acrylate homo- and copolymerizations are often carried out at high temperatures. Some of the advantages are fast polymerization rates, reduced viscosities compared to lower temperatures, and access to low molar mass polymers [14]. However, at higher temperatures some elemental reactions that have only a minor impact on molar mass distributions and reaction rate in low temperature polymerizations become increasingly important. In particular the extent of β scission reactions and subsequent radical migration is increased at high temperature [9,10,12]. Moreover, acrylate monomers were reported to undergo thermally induced self-initiation. Various reports by Soroush, Rappe, Grady and coworkers deal with the self-initiation in alkyl acrylate polymerizations in bulk and in solution in a temperature range from 120 to 220 °C [15,16,17,18,19,20,21,22,23]. It was suggested that the initiation process consists of several steps. Firstly, two monomer molecules form a singlet diradical, which undergoes intersystem crossing and a triplet diradical is obtained. This triplet radical reacts with a monomer resulting in two monoradicals [23]. The overall or apparent reaction involves three monomer molecules and is suggested to be second order. This apparent reaction may be expressed by Equation (1) [19]:

3 M → MM* + M*(1)

Intersystem crossing is supposed to be the slowest step [20,21].

To derive the kinetic coefficient of the self-initiation reaction the polymerizations were modelled [15,16,17]. To the best of our knowledge so far no clear picture of self-initiated polymerizations emerges. One reason may be seen in the fact that kinetic coefficients for some of the elemental reactions affecting the radical type and concentration, e.g., backbiting or β scission, are still under debate. Further, there are experimental challenges associated with carrying out the highly exothermic polymerizations under isothermal conditions [24]. In addition, it was suggested that the solvent has a significant impact on the polymerization rate and molar masses obtained [17].

Since the initiation process is second order with respect to monomer concentration it is important to monitor the monomer concentration throughout the reaction in order to obtain more information. Applying in-line monitoring techniques during the polymerization has several advantages: kinetic information are available over an extended conversion range with high time resolution from a limited number of experiments. Moreover, avoiding sampling throughout the reaction avoids any disturbances, e.g., due to the introduction of oxygen, variations in temperature, or sampling a non-representative sample could alter the composition of the reaction mixture. Various types of in-line monitoring were reported so far [25,26,27]. FT-NIR provides direct access to conversion vs. time data for homogeneous phase reactions due to the fact that the first overtone of the olefinic CH stretching vibration is rather intense and not overlapping with other vibrations for a large variety of monomers. Moreover, the method may be applied to a wide range of reaction conditions, e.g., varying temperature as well as solvent type and content [25,28,29,30,31,32,33,34]. Another very interesting approach is monitoring the polymerization via DSC, as already reported for a number of acrylate and methacrylate monomers [35,36,37,38,39,40,41]. Knowing the standard polymerization enthalpy the rate of polymerization may be calculated throughout the reaction.

The objective of this contribution is to get a better understanding of self-initiated polymerizations. For this purpose polymerizations were monitored in-line via FT-NIR and DSC. In order to perform isothermal BA radical polymerizations at high temperatures effective heat removal from the polymerizing sample is essential. Two approaches were pursued that fulfill this requirement: Firstly, a set of bulk polymerizations was carried out in DSC crucibles in a temperature range from 80 to 130 °C. Secondly, BA solution polymerizations were performed in dioxane, which allows for extension of the temperature range to 180 °C. Both methods provide the rate of polymerization as a function of monomer conversion. Further, solution polymerizations at 130 °C were carried out with different solvents in order to identify the impact of the solvent nature on the rate of polymerization.

## 2. Materials and Methods

### 2.1. Materials

The monomer n-butyl acrylate (BA, ≥99%, stabilized with 10–60 ppm monomethyl ether hydroquinone, Sigma Aldrich, Darmstadt, Germany) was distilled at 5 mbar to remove the inhibitor. The solvents 1,4 dioxane (99.8%, water free, Sigma Aldrich), 2-octanone (≥98%, Sigma Aldrich), toluene (99.9%, Sigma Aldrich, Darmstadt, Germany), xylene (technical mixture of isomers, VWR Elements) and mesitylene (99%, Acros Organics, Schwerte, Germany) were used as received. Tetrahydrofuran (THF, 99%, Grüssing, Filsum, Germany) was employed as eluent for size-exclusion chromatography (SEC). Hydroquinone (99%, Riedel) dissolved in methanol (98.5%, CG Chemikalien, Hannover, Germany) was used to stop the polymerization.

### 2.2. Characterization

FT-NIR spectra were recorded on a Bruker Vertex 70 spectrometer using a halogen lamp, a Si-coated CaF2 beamsplitter, and a liquid N_2_ cooled InSb detector. The spectra were recorded with a resolution of 2 cm^−1^. For slow reactions every minute a spectrum was taken using 20 scans. For faster reactions, especially at temperatures above 140 °C, the time interval was reduced to obtain a good time resolution. Therefore, four or six spectra were recorded per minute and the number of co-added scans reduced to five or three, respectively.

DSC measurements were carried out using a Mettler Toledo DSC1/500658/200W STAR^e^-system, which uses a FRS-5 sensor. For operation of the instrument and data evaluation the STAR^e^-Software 9.20 is employed. Polymerizations were performed in crucibles suited for elevated pressure (Mettler Toledo 26929).

### 2.3. Polymerization with In-Line DSC Measurement

Polymerizations with in-line DSC measurement were carried out in bulk at temperatures between 80 °C and 130 °C in crucibles allowing for pressures up to 20 bar. The monomer was purged with nitrogen to remove oxygen prior to filling the crucibles. For the reactions a precisely known mass of BA between 12.0 mg and 18.4 mg was used. The exact mases are listed in Table S1 of the Supporting Information. The heating-rate was set to 20 K·min^−1^. After reaching the selected reaction temperature the sample was held under isothermal conditions.

### 2.4. Polymerization with In-Line FT-NIR Measurement

BA polymerizations in solution were carried out in an optical high-pressure cell equipped with two sapphire windows allowing for quantitative in-line FT-NIR spectroscopy at temperatures up to 180 °C resulting in slightly elevated pressure of at most 10 bar. The reaction cell [25] was heated to the desired reaction temperature and vacuum was applied to remove oxygen. The reaction mixture consisting of monomer and solvent was purged with nitrogen to replace oxygen. The reaction cell was positioned in the sample compartment of the FT-IR/NIR-spectrometer (Bruker Vertex 70). The background spectrum was recorded with the empty reaction cell at the polymerization temperature. Then, the reaction mixture was introduced into the cell with a syringe mounted on the valve of the cell, and subsequently, the valve was closed. Recording of NIR-spectra was started directly after addition of the reaction mixture. The polymerizations were stopped and the reaction mixture removed from the cell with a syringe. A solution of 4 mg hydroquinone in 1 mL of methanol was added to stop the polymerization. At room temperature residual BA and the solvent were evaporated.

## 3. Results and Discussion

Since radical polymerizations of BA are exothermic, carrying out polymerizations under isothermal conditions is challenging. This is particular true for high-temperature reactions devoted to investigations into the self-initiation process. In addition, due to the rather high volatility of BA and the solvents used inside a closed reaction cell slightly elevated pressures of at most 10 bar occur at high temperatures. Thus, the reactions were carried out in either DSC crucibles designed for elevated pressure or in optical high-pressure cells allowing for in-line FT-NIR monitoring. It is well known that a slightly enhanced pressure up to at most 50 bar has a negligible impact on radical polymerizations of liquid monomers [42,43,44].

### 3.1. BA Polymerizations with In-Line DSC Monitoring

**Figure 1:**
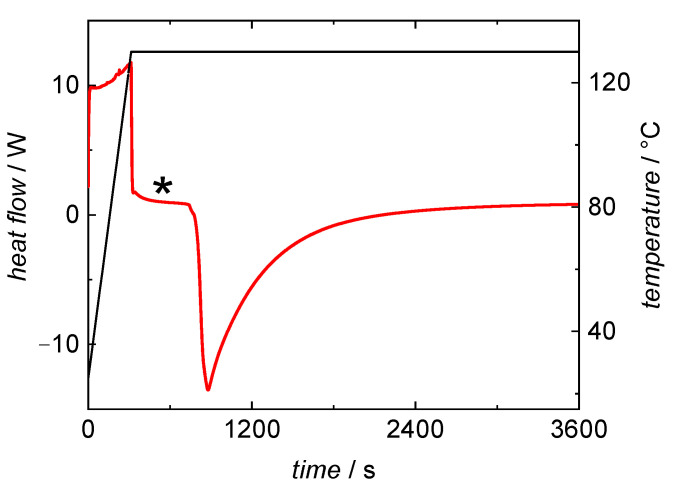
DSC curve from a BA polymerization at 130 °C under isothermal conditions. The heat flow curve (red) and temperature profile (black) were recorded while heating to the desired temperature and during the bulk polymerization. * marks the baseline used for data evaluation.

In-line DSC monitoring of BA bulk polymerizations was chosen, because kinetic information should be accessible with the knowledge of the enthalpy of polymerization. To extract kinetic data from the DSC curve the endothermic area resulting from the heating process of the probe needs to be well separated from the exothermic area being due to the polymerization reaction. A typical DSC curve recorded during a BA bulk polymerization at 130 °C is shown in Figure 1. The red curve shows the heat flow, the black data represents the temperature profile during the measurement. In the initial phase heating from room temperature to the reaction temperature occurs. This is an endothermal process, and therefore, the heat flow is positive. When the probe reaches the selected temperature the heat flow begins forming a baseline, marked with an asterisks in Figure 1. Once the exothermal self-initiated polymerization begins the heat flow is below the baseline. The measured heat flow is directly corresponding to the reaction rate of the polymerization.

Figure 1 indicates that the reaction does not start immediately after reaching the selected temperature. This delay results in well-separated endothermic and exothermic areas. At higher reaction temperatures the separation between heating-period and the start of the reaction becomes less pronounced and reliable data evaluation is difficult. Therefore, the method was not applied to polymerizations above 130 °C.

The overall reaction rate, *r*_br_, is calculated according to Equation (2) with the exothermal heat-flow, d(∆*H*)/d*t*, resulting from the polymerization reaction, *c*_BA,0_, and the standard reaction enthalpy ∆*H*_BA_^0^ = −79.84 kJ·mol^−1^ taken from [45].

(2) $$r_{\mathrm{br}}=-\frac{d(\Delta H)}{dt}\cdot \frac{c_{\mathrm{BA},0}}{\Delta H_{\mathrm{BA},\;x=1}},$$

with ∆*H*_BA, x = 1_ being calculated from the BA mass in the sample, *m*_BA_, molar mass of BA, *M*_BA_, and ∆*H*_BA_^0^ according to ∆*H*_BA, x = 1_ = ∆*H*_BA_^0^·*m*_BA_/*M*_BA_. ∆*H*_BA, x = 1_ refers to the enthalpy at full conversion Integration of the exothermic area of the DSC curve allows for calculation of the monomer conversion at a given time, *x*_t_, according to Equation (3). Thus, conversion vs. time data is accessible.

(3) $$x_{t}=\int_{t=0}^{t}\frac{d(\Delta H)}{dt}\cdot \frac{1}{\Delta H_{\mathrm{BA},\;x=1}},$$

**Figure 2:**
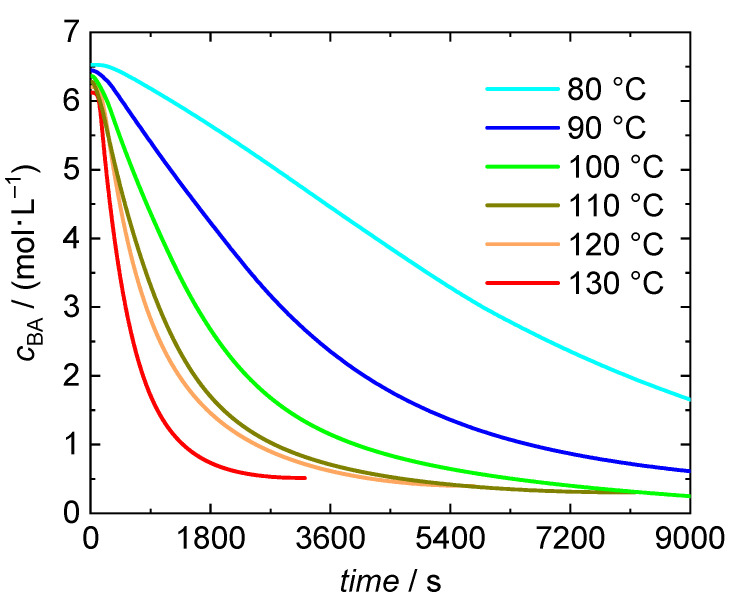
Concentration vs. time data for BA bulk polymerizations at temperatures between 80 °C and 130 °C with in-line DSC measurement. The timestamp with value 0 is set at the time at which the polymerizations starts. The integration of the DSC data starts at this time, too.

The conversion data is used to calculate BA concentrations as a function of time, which is given in Figure 2 for bulk polymerizations at temperatures ranging from 80 to 130 °C. It is remarkable to note that even at 80 °C self-initiation is able to start the polymerization. All polymerizations yield high BA conversions of at least 92.8%. A systematic variation with temperature is not found. Experimental details and conversions reached are provided in Table S1 of the Supporting Information. Temperature dependent densities of monomer and polymer were considered for the calculation of concentration from conversion data.

**Figure 3:**
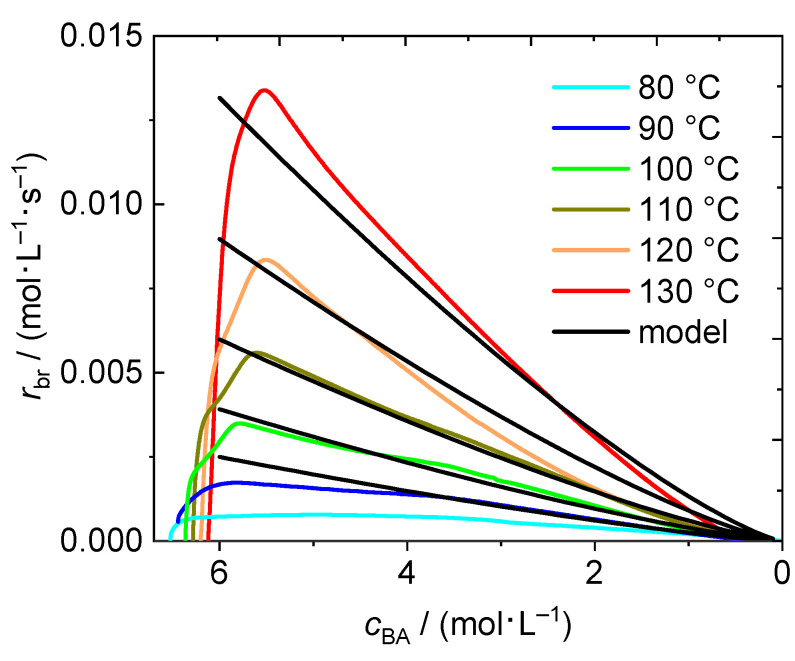
Rate of polymerization as a function of the actual BA concentration in the reaction mixture for the temperatures between 80 °C and 130 °C. Data resulting from the model calculation with Equation (4) is shown in black.

In Figure 3 *r*_br_ is shown as a function of the actual BA concentration in the system, *c*_BA_, to facilitate comparison with the results from solution polymerizations in the remainder of the work. The x-axes is plotted from high to low values, because the highest concentration refers to time zero. Interestingly, the maximum of the reaction rate is not observed at the highest concentration. This observation is in line with the finding that the minimum in the heat flow curve in Figure 1 is not reached directly at the start of the reaction, but shortly after the beginning of the polymerization. During this time interval some monomer has already reacted and monomer concentration is below the initial concentration, the monomer conversion is no longer zero. The maximum of the reaction rate is reached at a concentration of at least 6 mol·L^−1^ for temperatures in the range from 90 °C to 130 °C. Even at a temperature of 80 °C the self-initiated polymerization takes place, however, the rate is very small. Approximately 80% of conversion were reached only after 400 min. Consequently, the heat flow originating from the exothermal reaction is always very low and the data obtained at 80 °C is not included in the description of the overall rate of polymerization.

The DSC data from temperatures between 90 and 130 °C is used to build a facile model for the description of the reaction rate in a self-initiated bulk polymerization of BA. *r*_br_ is calculated according to Equation (4) with the overall rate coefficient, *k*_eff,b_, and the actual BA concentration, and the reaction order n. The reaction order is introduced since BA serves as initiator and monomer.

(4) $$r_{\mathrm{br}}=-\frac{dc_{\mathrm{BA}}}{dt}=k_{\mathrm{eff},b}\cdot c_{\mathrm{BA}}^{n},$$

To account for the temperature dependence of *r*_br_ the logarithmic form of Equation (4) is considered and *k*_eff,b_ is expressed by the Arrhenius relation ln *k*_eff,b_ = ln*A* – *E*_a_/R*T* resulting in Equation (5).

(5) $$\ln r_{\mathrm{br}}=\ln A-\frac{E_{a}}{RT}+n\ln c_{\mathrm{BA}}^{},$$

with the pre-exponential factor, *A*, and the activation energy of *k*_eff,b_, *E*_a_, the universal gas constant, R, and temperature *T*.

For analysis of the DSC measurements given in Figure 3 the experimentally derived curves were interpolated linearly leading to the same number of data points for each experiment independent of the reaction temperature. In order to avoid contributions from the heating period at the beginning of the reaction, only data between 5.9 mol·L^−1^ and 2.0 mol·L^−1^ is considered rather than using the entire data set. The lower limit was set to 2.0 mol·L^−1^ since the heat flow is rather small for *c*_BA_ below 2.0 mol·L^−1^ leading to a poor signal to noise ratio. An increment of 0.1 mol·L^−1^ was chosen for the interpolation. The resulting data sets consist of 200 data points. These experimentally-derived data points were used for the parameterization of the model. For each data point a tuple consisting of three values (ln[*c*_BA_], ln[*r*_br_], 1/R*T*) was calculated. Using the program ‘R’ (The R Project for Statistical Computing [46]) a multiple linear regression using the model Equation (6) is used. With this Equation the coefficients *a*_0_, *a*_1_ and *a*_2_ of the model were determined and listed in Table 1.

(6) $$y^{\prime }=\ln r_{\mathrm{br}}=a_{0}-a_{1}\cdot \frac{1}{RT}+a_{2}\cdot \ln c_{\mathrm{BA}}^{},$$

The coefficients from Table 1 were used to calculate d*c*_BA_/d*t* as a function of time for temperatures ranging from 90 to 130 °C. This data is plotted against *c*_BA_ in Figure 3 for comparison with the experimental data. A good agreement between modeled and experimentally derived data is found. Therefore, the simple model may be used to estimate the reaction rate of thermally self-initiated BA polymerizations in advance when considering a temperature range between 90 °C and 130 °C.

The finding that data from bulk polymerizations at four different temperatures may be represented by this simple Equation, which does not explicitly account for diffusion control of the termination reaction, is very surprising. To explain this finding the following aspects reported need to be kept in mind. It is known that the chain-length averaged termination rate coefficients, <*k*_t_>, in BA systems do not vary to a great extent with monomer conversion at a given chain length [47]. This point is addressed in more detail below.

Moreover it is instructive to consider the value of the coefficient *a*_2_, which resembles the reaction order. A value slightly higher than one agrees with the fact that the order of the propagation reaction is one and that monomer is also involved in the initiation reaction. The parameters *a*_0_ and *a*_1_ represent ln*A* and *E*_a_, respectively, of the overall rate coefficient *k*_eff,b_, which contains contributions of all elemental reactions on the rate. Thus, the physical meaning of these coefficients cannot easily be evaluated

### 3.2. BA Polymerizations with In-Line FT-NIR Monitoring

Since the experiments with DSC monitoring were limited to a maximum temperature of 130 °C additional polymerizations were carried out with FT-NIR monitoring to expand the temperature range. Since bulk polymerizations were too fast for an appropriate time resolution of the recorded spectra the reactions were carried out in solution. For this purpose the polymerizations were performed in optical high-pressure cells. Besides being suited for elevated pressure the cells are advantageous with respect to temperature control, because the stainless steel cell body has a high heat conduction coefficient. The cell mass is 5.34 kg, while the mass of the reaction mixture is only around 2 g. Thus, carrying out the polymerizations in this special reaction cell promotes a nearly constant temperature. A thermocouple placed directly at the reaction volume showed that the temperature varied by at most 0.5 °C, both after addition of the reaction mixture to the heated cell and during the polymerization. Further, the optical cell allows for in-line FT-NIR spectroscopy and, thus, monitoring of monomer conversion during the reaction [25,34].

**Figure 4:**
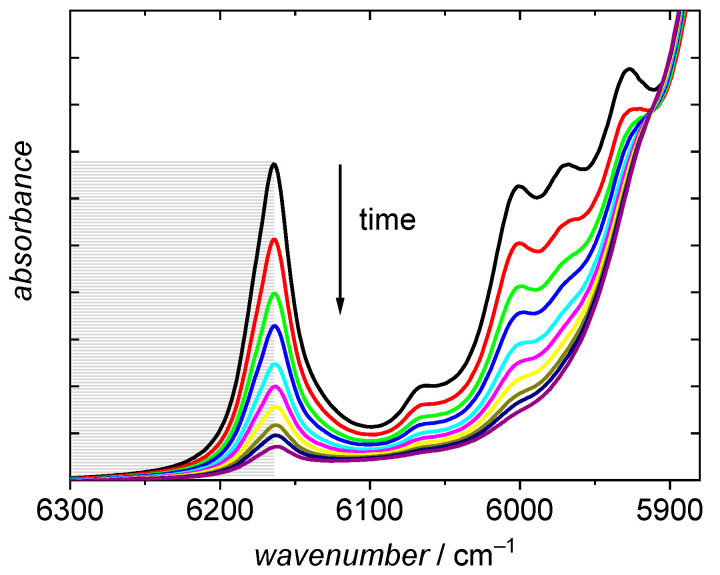
NIR spectra recorded during a BA polymerization in dioxane. Temperature and initial monomer concentration are 150 °C and 3.69 mol·L^−1^, respectively. The final spectrum refers to a reaction time of 30 min.

To estimate monomer conversion the absorption band at 6166 cm^−1^ [48], which is assigned to the first overtone of the olefinic C-H stretching vibration, was employed. The half peak is integrated from the peak maximum at 6166 cm^−1^ to 6300 cm^−1^ using a horizontal baseline at the latter wavenumber as illustrated by the grey area in Figure 4. To minimize contributions from absorptions assigned to the saturated C-H stretching vibrations below 6166 cm^−1^ the half peak rather than the full peak is integrated. During polymerization the fraction of monomer is lowered and consequently, the peak intensity decreases.

Monomer conversion as a function of time is calculated from the integral of the first NIR spectrum recorded directly after inserting the reaction mixture into the optical high-pressure cell at zero conversion, *A*_0_, and the integral *A*_t_ obtained from the spectrum recorded at a given time *t* according to Equation (7).

(7) $$x_{t}=\frac{A_{0}-A_{t}}{A_{0}},$$

The BA concentration as a function of time is calculated according to Equation (8), with the initial BA concentration, *c*_BA,0_.

(8) $$c_{\mathrm{BA},t}=\left(1-x_{t}\right)\cdot c_{\mathrm{BA},0},$$

**Figure 5:**
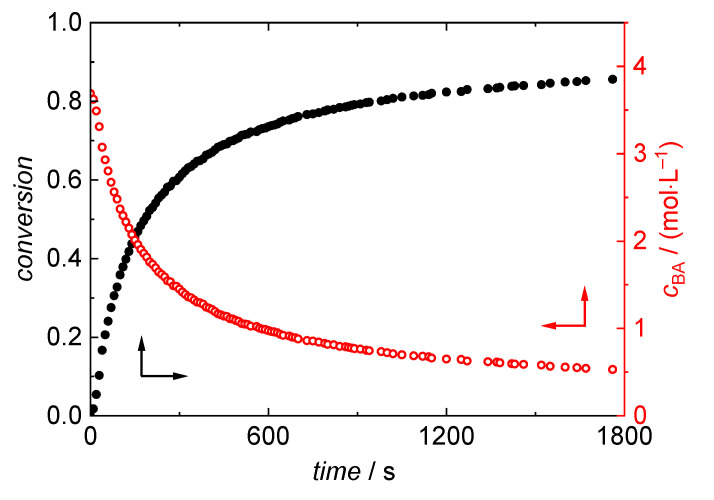
Conversion (black) and *c*BA (red) derived from NIR spectra for a polymerization in dioxane at 150 °C with an initial BA concentration of 3.69 mol·L^−1^.

*c*_BA,0_ is calculated under the assumption of ideal mixing of monomer and solvent using the BA density at room temperature. *c*_BA_,_0_ is considered invariant with temperature, since the reaction mixture kept at room temperature is quickly introduced into the fixed volume of the reaction cell, which is completely filled with the reaction mixture. As an example, the variation of BA concentration and conversion with time is plotted in Figure 5 for a polymerization carried out at 150 °C in dioxane with an initial BA concentration of 3.69 mol·L^−1^. The conversion is steadily increasing up to around 86% at the end of the reaction after one hour. As the conversion is increasing, the concentration decreases to 0.53 mol·L^−1^. Table 2 gives BA conversions and residual BA concentrations for polymerizations carried out for temperatures ranging from 120 °C to 180 °C.

It is evident that the peak assigned to the olefinic CH stretching vibration in Figure 4 is not fully disappearing. Consequently, full conversions are not calculated according to Equation (7). The observation that the olefinic peak at 6166 cm^−1^ does not fully disappear at high reaction times may be explained by two points. The first point is specific for high temperature polymerizations: As a result of β scission reactions BA macromonomers are formed, which were identified by ESI-MS analyses [10,12] These macromonomers cannot be distinguished from BA monomer in the NIR spectra and consequently a lower conversion is calculated from the NIR spectra. Secondly, contributions from the absorptions of saturated C-H stretching vibrations may overlap with the absorption due to the olefinic stretching vibration. The apparent conversion in Figure 4 is 86%. Both contributions mentioned are the stronger the higher the monomer conversion. Thus, the discussion of the kinetics at low monomer conversions is not hampered by this point.

At rather low temperatures from 120 to 140 °C the reaction time was set to one hour. As expected, with increasing temperature *x* is enhanced as well. At higher temperatures the reaction time was reduced because of the strongly enhanced polymerization rate. For example, at 180 °C a conversion of 86% is reached already after 10 min.

**Figure 6:**
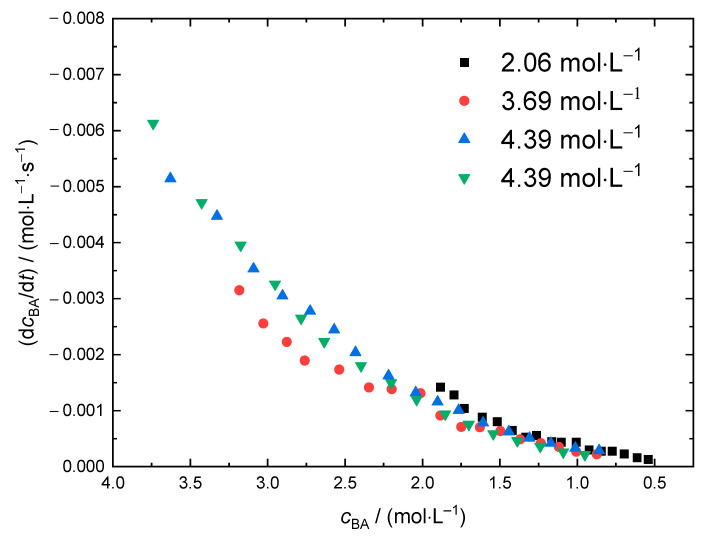
d*c*_BA_/d*t* as a function of the current monomer concentration derived from NIR spectra recording during polymerizations at 120 °C. The legend gives the initial BA concentrations, *c*_BA,0_.

For further investigations into the thermal self-initiation of BA, the rate of monomer consumption d*c*_BA_/d*t* is plotted against the current BA concentration. Thus, polymerizations carried out at different solvent content may be compared conveniently. As an example, the data obtained at 120 °C are given in Figure 6. The initial monomer concentrations varied between 4.39 mol·L^−1^ and 2.06 mol·L^−1^, that corresponds to molar ratios *x*_BA_ of BA to solvent in the range from 0.5 to 0.2. The reproducibility of the data obtained from two polymerizations at *c*_BA,0_ = 4.39 mol·L^−1^ is very good. It is interesting to note that the data from polymerizations with significantly different initial BA concentrations differs only slightly. At first sight this finding is rather surprising, because a lowering of the monomer concentration does not only result in shorter polymer chains and promotes transfer to solvent. More importantly, the intramolecular transfer reaction, the so-called backbiting reaction, yielding tertiary propagating radicals becomes more probable as well. Since the propagation rate coefficients of these tertiary radicals are by three orders of magnitude lower than the propagation rate coefficients of the chain-end radicals, a decrease in *r*_br_ might have been expected. In addition, it may be anticipated that different initial monomer concentrations lead to different viscosities throughout the polymerizations, which should have an influence on the diffusion-controlled termination reaction. This point will be discussed later in more detail.

**Figure 7:**
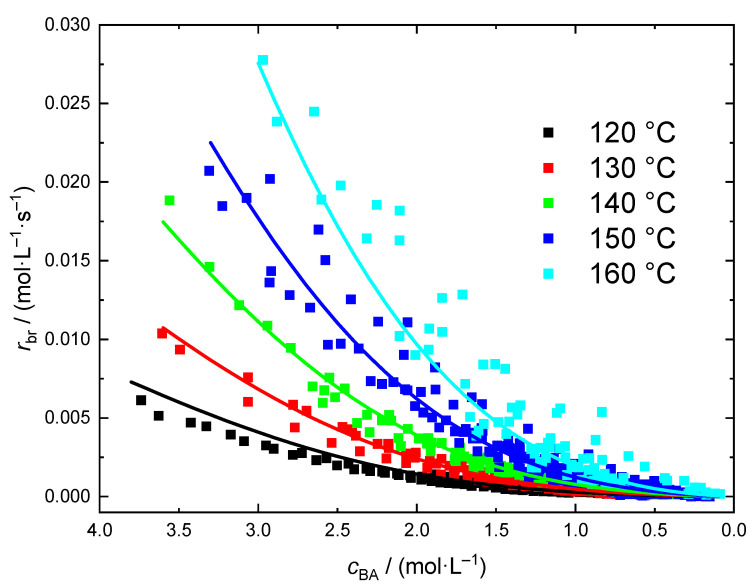
*r*_br_ data obtained for polymerizations ranging from 120 to 160 °C. The lines were calculated according to Equation (11) with the parameter set given in Table 3.

The rate data from independent polymerizations at a given temperature and different *c*_BA,0_ is shown in Figure 7. As expected a significant impact of the temperature is seen. Due to the exothermicity of the polymerization the initial BA concentrations had to be lowered with increasing temperature. The complete list of experiments with temperature and *c*_BA,0_ is given in Table S2 in the Supporting Information. In addition, molar mass information is contained.

At all temperatures polymerizations with at least two different *c*_BA,0_ were carried out. In all cases the data from experiments with different *c*_BA,0_ coincide nicely. Since the kinetic data for the different *c*_BA,0_ overlap it was tested whether a rather simple kinetic model similar to the bulk case may represent the entire rate data set for polymerizations in dioxane. The analysis of the rate data in Figure 7 is based on Equation (9).

(9) $$r_{\mathrm{br}}=k_{\mathrm{eff},s}\cdot c_{\mathrm{BA}}^{n}\cdot \exp(l\cdot c_{\mathrm{BA}}^{}+m\cdot c_{\mathrm{BA}}^{2}),$$

As detailed above for the bulk polymerizations an overall rate coefficient, here *k*_eff,s_, the reaction order n, and the actual BA concentration *c*_BA_ at a certain time is introduced. Contrary to the bulk system additional parameters *l* and *m* are used, which account for the impact of the solvent. For example, *l* and *m* are supposed to account for effects due to non-ideal mixing on the molecular level. To include the temperature dependence of the kinetic coefficient *k*_eff,s_ is expressed by its Arrhenius relation and the logarithmic form of Equation (10) is considered:

(10) $$\ln r_{\mathrm{br}}=\ln A-\frac{E_{a}}{RT}+n\cdot \ln c_{\mathrm{BA}}^{}+l\cdot c_{\mathrm{BA}}^{}+m\cdot c_{\mathrm{BA}}^{2},$$

(11) $$y^{\prime }=\ln r_{\mathrm{br}}=a_{0}-a_{1}\cdot \frac{1}{RT}+a_{2}\cdot \ln c_{\mathrm{BA}}^{}+a_{3}\cdot c_{\mathrm{BA}}^{}+a_{4}\cdot c_{\mathrm{BA}}^{2},$$

Firstly, it is assumed that the presence of the solvent does not affect the reaction order. Thus, the value of 1.28 determined for *a*_2_ on the basis of the bulk polymerization data is used for the solution polymerizations, too. For parametrization of Equation (11) the entire data set (730 individual points) depicted in Figure 7 is applied. For each data point a tuple consisting of four values (ln[*c*_BA_], ln[*r*_br_], 1/R*T*, *c*_BA_) was calculated and the program ‘R’ was used for a multiple linear regression according to the model Equation (11). The coefficients *a*_0_, *a*_1_, *a*_3_, and *a*_4_ of the model listed in Table 3 were determined. The full lines in Figure 7 were calculated according to Equation (11) with the coefficients listed in Table 3. It is remarkable to note that a rather facile model based on 5 parameters provides a good representation of the variation of *r*_br_ with temperature and monomer concentration in the case of BA polymerization in dioxane.

**Figure 8:**
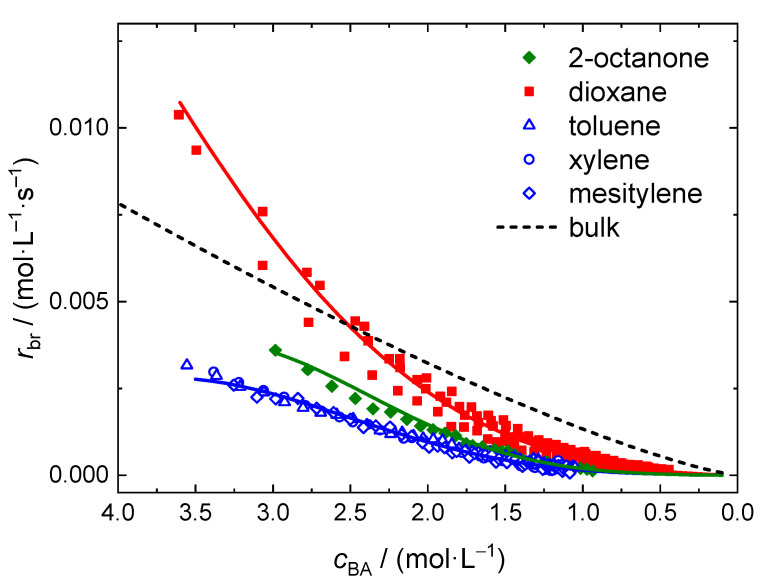
*r*_br_ data obtained for polymerizations at 130 °C in the indicated solvents with *x*_BA_ = 0.5. The full lines were calculated according to Equation (13) with the parameter set given in Table 5 and the dashed line with Equation (6) and parameters from Table 1.

Finally, a number of polymerizations at 130 °C was carried out in several additional solvents. 2-octanone and xylene were selected since they have a rather high boiling temperature and are technically relevant, respectively. Generally, xylene is provided as an isomeric mixture in technical quality. In order to use aromatic solvents of high purity toluene and mesitylene were used as well. Again, the reactions were carried out with in-line FT-NIR monitoring of BA conversion. In all cases the molar ratio of monomer to solvent is 0.5. The NIR spectra are similar to the ones obtained for reactions in dioxane. However, the aromatic solvents show significant absorptions, which overlap with the peak assigned to the olefinic stretching vibration at 6166 cm^−1^. As detailed for MMA polymerizations in toluene the contributions of the aromatic solvents were subtracted from the NIR spectra prior to integration [34]. The resulting NIR spectra were integrated as above-described and the rate data is presented in Figure 8. It is evident that the aromatic solvents lead to a significant lower polymerization rate. In addition, no significant differences are found for toluene, xylene, and mesitylene as solvents. The rate of polymerizations carried out in 2-octanone is slightly higher than for the aromatic solvents, but still significantly lower than in dioxane. The dashed line represents the data obtained for bulk polymerizations and was calculated according to Equation (5) with the coefficients given in Table 2.

Since the solvent content for the polymerizations presented in Figure 8 is the same it may be expected that the change in viscosity throughout the reaction is rather similar. Thus, it is suggested that the significantly higher *r*_br_ values for dioxane as solvent are not due a solvent influence on the diffusion-controlled termination reaction. A significantly different impact of either dioxane or the other solvents on the extent of elemental reactions such as backbiting, migration, or β scission is considered to be unlikely [12]. Further, the chain transfer to solvent rate coefficient for BA polymerizations is not significantly different for the solvents used here [49]. From literature it is well known that the presence of organic solvents may affect the propagation rate coefficient, *k*_p_, considerably [50,51,52,53,54,55,56]. Previously, an enhancement in propagation rate coefficients was reported for systems in which the carbonyl group of the monomer was engaged in direct hydrogen bonding [54,55]. For example, polymerizations of butyl methacrylate in butanol at 70 °C and 25% of monomer are associated with *k*_p_ values being 35% higher than the corresponding bulk value. The difference is lower at higher temperatures. For systems without any specific interactions between monomer and solvent, as for BA polymerizations in the aromatic solvents or in 2-butanone, only a minor solvent influence on *k*_p_ was reported [51,53,56]. Typically *k*_p_ was changed by 10 to 20% due to the presence of the solvent. To the best of our knowledge propagation rate coefficients were not yet reported for (meth)acrylate polymerizations in dioxane.

In order to estimate or judge the solvent influence on chemical reactions various kinds of solubility parameters were introduced [57]. Rather than using parameters that account for the solvent influence in a single value it may be instructive to consider parameters that distinguish between different contributions, e.g., such as dispersion forces, polarity, solvent basicity, or hydrogen bonding ability. Along this line the strong influence of ionic liquids on *k*_p_ was explained with Kamlet Taft parameters [58]. Here, Hansen solubility parameters are considered, which differentiate between contributions from non-polar interaction (dispersion), δ_D_, polar interaction, δ_P_, and hydrogen bonding interaction, δ_H_ [59]. Published data for toluene, xylene, and dioxane are listed in Table 4. Data for 2-octanone is not available. Data for acetone and 2-butanone were included to allow for a comparison with the other solvents. Firstly, it is seen that the values for toluene and xylene are rather similar. The individual values for dioxane are higher than the corresponding data for the aromatic solvents. The largest difference is seen for δ_H_ representing hydrogen bonding interactions. Thus, in the case of dioxane as solvent it may be anticipated that contributions from hydrogen bonding may enhance *k*_p_ slightly. It is to be expected that the increase in *k*_p_ is not comparable to the above-mentioned butyl methacrylate/butanol system, because the dioxane concentration is significantly lower and hydrogen bonding between an OH and a carbonyl group is expected to be stronger. Thus, it is anticipated that a potential increase in *k*_p_ cannot account for the significant higher *r*_br_ values.

Based on the data for acetone and 2-butanone it is expected that polar and hydrogen bonding interactions of 2-octanone are lower than for 2-butanone. Thus, δ_H_ is expected to lie between the values for 1,4 dioxane and the aromatic solvent. The rate data for polymerizations in 2-octanone are between the data for the aromatic solvents and dioxane. The findings suggest that the ability of the solvent to engage in H-bonding may contribute to the observed differences in polymerization rate only to a small extend.

Since it is unlikely that dioxane affects propagation, the various chain transfer reactions or termination to a large extend, dioxane may have an accelerating impact on the self-initiation reaction. To test this suggestion it was investigated at which temperature considerable polymerization is observed. BA polymerization in dioxane is already observed at temperatures as low as 70 °C, while bulk polymerizations require 80 °C to obtain considerable conversion. Similarly, other acrylates showed self-initiation in the presence of dioxane 20 to 30 °C below the temperature of the bulk system. To exclude that peroxides potentially being contained in dioxane are responsible for this finding, freshly distilled dioxane was used, too. Still, BA polymerization in dioxane was observed already at 70 °C. Considering the complex self-initiation mechanism, which involves a singlet diradical that undergoes intersystem crossing to form a triplet radical, it may be envisioned that dioxane interacts favorable with the transition state structures resulting in faster self-initiation.

While the discussion indicates that the significantly different rate data for polymerizations in dioxane appears reasonable, it goes without saying that a detailed investigation into BA polymerizations in dioxane is required to disclose the origin of the dioxane influence on *r*_br_.

Previously it was reported that the chain-length averaged termination rate coefficient <*k*_t_> determined from bulk polymerizations is only slightly reduced with conversion [47]. Moreover, it was demonstrated that the chain length dependence is much weaker as for example for styrene or methyl methacrylate. For chain length higher than 20 only a very modest reduction in <*k*_t_> was observed with increasing chain length [47]. Still, the slightly higher polymerization rate of polymerizations in bulk compared to reactions in aromatic solvents or 2-octanone may be due to a higher viscosity compared to the solution polymerizations at a given BA concentration, which is expected to lead to some lowering of the diffusion-controlled termination rate and consequently an enhanced polymerization rate.

Similar to the reactions in bulk and in solution for various temperatures, the rate data from polymerizations in 2-octanone at 130 °C and the combined data set of the polymerizations in the aromatic solvents are fit according to Equation (13). Since all experiments in Figure 8 were carried out at 130 °C the term representing the temperature dependence in Equation (11) is not required. Parameters *a*_0_ and *a*_1_ from Equation (11) are combined to a single parameter *a*_01_. Again, the reaction order of 1.28 determined for the bulk polymerizations and introduced for the description of the data from polymerizations in dioxane at 120 to 160 °C was used. The values obtained for the coefficients *a*_01_, *a*_3_, and *a*_4_ are listed in Table 5.

(12) $$\ln r_{\mathrm{br}}=k_{\mathrm{eff},s}+n\cdot \ln c_{\mathrm{BA}}^{}+l\cdot c_{\mathrm{BA}}^{}+m\cdot c_{\mathrm{BA}}^{2},$$

(13) $$y^{\prime }=\ln r_{\mathrm{br}}=a_{01}+a_{2}\cdot \ln c_{\mathrm{BA}}^{}+a_{3}\cdot c_{\mathrm{BA}}^{}+a_{4}\cdot c_{\mathrm{BA}}^{2},$$

The full lines in Figure 8 were calculated according to Equation (13) with the parameters from Table 5. Again a very good representation of the data is reached.

The models introduced for bulk and solution polymerizations allow for a generalization of the experimental data. They provide *r*_br_ as a function of the actual monomer concentration at each instant of the polymerization. It is stressed that the coefficient *k*_eff_ in Equations (4), (9), and (12) does not refer to an individual elementary reaction. It is evident from the experimental results that the solvent has a significant impact on the polymerization rate. Due to the complex reaction mechanism an assignment of the solvent influence to distinct elementary reactions is not possible at this stage. Thus, in future BA polymerizations in bulk and in the various solvents will be modeled via Monte Carlo methods to estimate the unknown kinetic coefficients.

## 4. Conclusions

BA polymerizations with thermal initiation of the monomer itself were investigated via in-line monitoring of the polymerization progress via DSC and FT-NIR. It is observed that bulk polymerizations may be initiated by the monomer itself already at 80 °C. DSC was feasible for following bulk polymerizations up to 130 °C. In order to expand the accessible temperature range to 180 °C solution polymerizations were carried out in dioxane, 2-octanone and various aromatic solvents. As expected the polymerization proceeds with the highest rate in the case of bulk reactions compared to polymerizations in 2-octanone or the aromatic solvents. Surprisingly, the polymerization in dioxane proceeds faster than in bulk at a given actual monomer concentration as long as *c*_BA_ is higher than 2.5 mol·L^−1^. For polymerizations in dioxane, 2-octanone, and in the aromatic solvents the rate of polymerization at a given *c*_BA_ decreases in the given order. In future studies it is important to investigate whether the strong enhancement in *r*_br_ in the presence of dioxane is due to its impact on the self-initiation reaction.

Although termination in free-radical polymerization is a diffusion-controlled reaction, the overall rate of polymerization data from bulk polymerizations may be represented as a function of actual monomer concentration in the system by a rather easy equation with three parameters accounting for the temperature dependence by an Arrhenius type approach. Similarly, rate data from polymerizations in solution with dioxane in a temperature range from 120 to 160 °C and for polymerizations at 130 °C in 2-octanone and the aromatic solvents may be described.

In the future the large experimental data set will be used for parameterization of kinetic coefficients of a full kinetic scheme for kinetic Monte Carlo simulations.

## Figures and Tables

**Table 1:** Estimated coefficients *a*_0_, *a*_1_, and *a*_2_ of the model represented by Equation (6) for bulk polymerizations ranging from 90 to 130 °C.

Coefficient	Value	Error
*a*_0_ (ln*A*)	8.47	0.20
*a*_1_ (*E*_a_)/(kJ·mol^−1^)	50.59	0.61
*a*_2_ (*n*)	1.28	0.03

**Table 2:** Monomer conversion, *x*, and residual BA concentration, *c*_BA,res_, determined for BA polymerizations in dioxane with *c*_BA,0_ = 3.69 mol·L^−1^ at the given temperatures, *T*, and reaction times, *t*.

*T*/°C	*x*	*c*_BA,res_/(mol·L^−1^)	*t*/s
120	0.82	0.78	3600
130	0.89	0.41	3600
140	0.92	0.31	3600
150	0.86	0.53	1800
160	0.82	0.67	600
170	0.83	0.62	600
180	0.86	0.51	600

**Table 3:** Estimated coefficients *a*_0_, *a*_1_, *a*_3_ and *a*_4_ of the model represented by Equation (11) for polymerizations in dioxane ranging from 120 to 160 °C. *a*_2_ (*n*) was set to 1.28.

Coefficient	Value	Error
*a*_0_ (ln*A*)	11.55	0.51
*a*_1_ (*E*_a_)/(kJ·mol^–1^)	67.52	1.80
*a*_3_ (*l*)/(L·mol^–1^)	1.04	0.08
*a*_4_ (*m*)/(L^2^·mol^–2^)	−0.10	0.02

**Table 4:** Hansen solubility parameters for the indicated solvents [59].

Solvent	δ_D_	δ_P_	δ_H_
toluene	18.0	1.4	2.0
xylene	17.6	1.0	3.1
1,4 dioxane	19.0	1.8	7.4
acetone	15.5	10.4	7.0
2-butanone	16.0	9.0	5.1

**Table 5:** Estimated coefficients a_01_, a_3_, and a_4_ of the model represented by Equation (13) for polymerizations in the indicated solvents with equimolar amounts of monomer and solvent at 130 °C. a_2_ was set to 1.28.

	Coefficient	Value	Error
2-octanone	*a*_01_	−10.40	0.16
	*a*_3_/(kJ·mol^−1^)	2.22	0.19
	*a*_4_/(kJ·mol^−1^)	−0.38	0.05
toluene, xylene, mesitylene	*a*_01_	−10.36	0.18
	*a*_3_/(kJ·mol^–1^)	1.85	0.19
	*a*_4_/(kJ·mol^−1^)	−0.29	0.04

## Data Availability

Not applicable.

## Supplementary Materials

The following are available online at https://www.mdpi.com/article/10.3390/polym13122021/s1, Table S1: Experimental details for bulk polymerizations, Table S2: List of reaction conditions of all polymerizations carried out in solution with dioxane, Table S3: List of reaction conditions of polymerizations carried out in solution with different solvents.
