# Supplementary material for: Self-Initiated Butyl Acrylate Polymerizations in Bulk and in Solution Monitored By In-Line Techniques
Source: Polymers (Basel). 2021 Jun 21;13(12):2021. doi: 10.3390/polym13122021 (PMC8233791; doi:10.3390/polym13122021)
Supplement: Supplementary file 1 [file polymers-13-02021-s001.zip › polymers-1226025-supplementary.pdf]

Supporting Information

Self-initiated butyl acrylate polymerizations in bulk and in solution monitored by in-line techniques

Jonas Mätzig, Marco Drache, Sabine Beuermann

Clausthal University of Technology, Institute of Technical Chemistry,

Arnold-Sommerfeld-Straße 4, 38678 Clausthal-Zellerfeld, Germany

Table S1: Experimental details for bulk polymerizations.

T / °C	m_{BA} / mg	ρ_{BA}[1] / (g·cm^{-3})	$\rho_{Poly(BA)}$[1] / (g·cm^{-3})	I / J	x	t (x=0.9) / s
80	18.431	0.836	0.984	11.45	0.997	12720
90	13.375	0.826	0.973	7.875	0.945	7950
100	13.708	0.816	0.962	8.382	0.982	4590
110	17.217	0.805	0.951	10.28	0.959	3524
120	12.357	0.795	0.940	7.277	0.945	3200
130	12.036	0.785	0.929	6.976	0.928	1410

Final monomer conversion at the end of the reaction, x, for bulk polymerizations with in-line DSC measurement at the indicated temperature, T, with the initial BA mass, m_{BA}, the time required for 90 % conversion, t (x=0.9), and the integral, I, of the exothermic DSC peak.

[1] Baruido, I.; Févotte, G. McKenna T.F. Density data for copolymer systems: butyl acrylate/vinyl acetate homo- and copolymerization in ethyl acetate, *Eur. Polym. J.* **1999**, *35*, 775-780, 10.1016/S0014-3057(98)00070-6.

Table S2: List of reaction conditions of all polymerizations carried out in solution with dioxane. M_p refers to the molar mass of the main peak of the molar mass distribution, M_n is the number average molar mass, D the dispersity, T the temperature, t reaction time, c_{BA} the initial monomer concentration and x_{BA} the initial molar ratio of monomer.

x_{BA}	$c_{BA,0}$ / (mol·L^{-1})	T / °C	t / min	M_n / (g·mol^{-1})	M_p / (g·mol^{-1})	D
0.20	2.06	120	60	$4.93 \cdot 10^3$	$3.21 \cdot 10^4$	5.0
0.30	2.92	120	60	$7.03 \cdot 10^3$	$4.85 \cdot 10^4$	6.3
0.40	3.69	120	60	$9.52 \cdot 10^4$	$7.43 \cdot 10^4$	8.5
0.50	4.39	120	60	$9.88 \cdot 10^3$	$9.18 \cdot 10^4$	10.8
0.20	2.06	130	60	$3.72 \cdot 10^3$	$1.74 \cdot 10^4$	4.7
0.30	2.92	130	60	$5.96 \cdot 10^3$	$4.24 \cdot 10^4$	6.3
0.40	3.69	130	60	$6.62 \cdot 10^3$	$5.40 \cdot 10^4$	7.9
0.50	4.39	130	60	$1.07 \cdot 10^4$	$7.13 \cdot 10^4$	7.1
0.20	2.06	140	60	$3.21 \cdot 10^3$	$1.36 \cdot 10^4$	4.4
0.30	2.92	140	60	$4.51 \cdot 10^3$	$3.30 \cdot 10^4$	5.7
0.40	3.69	140	60	$5.79 \cdot 10^3$	$4.22 \cdot 10^4$	6.1
0.50	4.39	140	60	$6.46 \cdot 10^3$	$5.20 \cdot 10^4$	7.2
0.10	1.10	150	30	$1.81 \cdot 10^3$	$3.47 \cdot 10^3$	2.7
0.20	2.06	150	30	$2.58 \cdot 10^3$	$1.03 \cdot 10^4$	3.7
0.30	2.92	150	30	$3.15 \cdot 10^3$	$1.46 \cdot 10^4$	4.2
0.40	3.69	150	30	$4.07 \cdot 10^3$	$3.26 \cdot 10^4$	5.6
0.10	1.10	160	30	$1.81 \cdot 10^3$	$2.27 \cdot 10^3$	4.7
0.20	2.06	160	10	$2.10 \cdot 10^3$	$6.06 \cdot 10^3$	3.3
0.30	2.92	160	10	$2.89 \cdot 10^3$	$1.23 \cdot 10^4$	3.9
0.40	3.69	160	10	$3.78 \cdot 10^3$	$1.75 \cdot 10^4$	4.8
0.10	1.10	170	30	$1.46 \cdot 10^3$	$2.04 \cdot 10^3$	3.1
0.20	2.06	170	10	$1.83 \cdot 10^3$	$3.46 \cdot 10^3$	2.8
0.30	2.92	170	10	$2.25 \cdot 10^3$	$7.16 \cdot 10^3$	3.5
0.40	3.69	170	10	$2.90 \cdot 10^3$	$1.33 \cdot 10^4$	4.4
0.10	1.10	180	30	$1.32 \cdot 10^3$	$1.71 \cdot 10^3$	1.7
0.20	2.06	180	10	$1.91 \cdot 10^3$	$3.21 \cdot 10^3$	2.3
0.30	2.92	180	10	$1.95 \cdot 10^3$	$4.24 \cdot 10^3$	3.4
0.40	3.69	180	10	$2.53 \cdot 10^3$	$9.75 \cdot 10^3$	4.0

Table S3: Reaction conditions of polymerizations carried out in solution with different solvents, including dioxane for comparison. M_p refers to the molar mass of the main peak of the molar mass distribution, M_n is the number average molar mass, D the dispersity, T the temperature, t reaction time, c_{BA} the initial monomer concentration and x_{BA} the molar ratio of monomer.

solvent	x_{BA}	$c_{BA,0}$ / (mol·L^{-1})	T / °C	t / min	M_n / (g·mol^{-1})	M_p / (g·mol^{-1})	D
dioxane	0.50	4.39	130	60	$1.07 \cdot 10^4$	$7.13 \cdot 10^4$	7.1
2-octanone	0.50	3.35	130	60	$9.11 \cdot 10^3$	$5.62 \cdot 10^4$	6.1
mesitylene	0.50	3.56	130	60	$8.03 \cdot 10^3$	$4.42 \cdot 10^4$	5.2
xylene	0.50	3.76	130	60	$7.75 \cdot 10^3$	$4.95 \cdot 10^4$	6.0
toluene	0.50	4.03	130	60	$1.02 \cdot 10^4$	$7.22 \cdot 10^4$	7.7
